# Wild Edible Plants: Ensuring Sustainable Food Security in an Era of Climate Change

**DOI:** 10.3390/foods14091611

**Published:** 2025-05-02

**Authors:** Mauri K. Åhlberg

**Affiliations:** Faculty of Educational Sciences, University of Helsinki, 00100 Helsinki, Finland; mauri.ahlberg@gmail.com

## 1. Introduction

Currently, there are more significant threats to food security compared to earlier decades because of the accelerating rate of climate change. Various geographical areas may become too hot, dry, rainy, or cold compared to previous decades. Ordinary crop plants may suffer and produce little or no harvest. However, many types of edible weeds (wild edible plants) can flourish under these conditions. The best wild edible plants are mostly weeds that humans have spread almost globally through agriculture. Italians have referred to them as alimurgic plants. They provide raw materials for healthy food even in times of struggle, war, and when there is a total loss of the ordinary harvest of cultivated plants. Fortunately, there has been an increasing amount of research on the uses of wild edible plants and their chemical constituents since the beginning of the 21st century. Research shows that many invasive plant species provide health-promoting food and ecosystem services. In this Special Issue, we invited researchers to explore these issues and present their research findings.

The following four articles were accepted for publication:

(1) Alrhmoun, M.; Sulaiman, N.; Pieroni, A. Phylogenetic Perspectives and Ethnobotanical Insights on Wild Edible Plants of the Mediterranean, Middle East, and North Africa. Foods 2025, 14, 465. https://doi.org/10.3390/foods14030465.
(2) Chen, Q.; Wang, M.; Hu, X.; Zhang, J.; Zhang, Q.; Xu, C.; Long, C. Traditional Knowledge and Efficacy Analysis of an Emerging Medicinal Food Plant: Disporopsis aspersa. Foods 2025, 14, 72. https://doi.org/10.3390/foods14010072.
(3) Casas, M.; Vallès, J.; Gras, A., Nutritional Properties of Wild Edible Plants with Traditional Use in the Catalan Linguistic Area: A First Step for Their Relevance in Food Security. Foods 2024, 13, 2785. https://doi.org/10.3390/foods13172785.
(4) Clemente-Villalba, J.; Burló, F.; Hernández, F.; Carbonell-Barrachina, Á.A. Potential Interest of Oxalis pes-caprae L., a Wild Edible Plant, for the Food and Pharmaceutical Industries. Foods 2024, 13, 858. https://doi.org/10.3390/foods13060858.

## 2. Discussion

The articles are listed in the reverse order of their publication. The Guest Editor presents and comments on the four articles based on his ten years of experience in meta-research on wild edible plants (WEPs). Åhlberg’s handbook is only one of the resources that his meta-research program has created [1]. His other publications can be found using a search engine like Google and the keywords <ORCID Mauri K. Åhlberg>. (ORCID is Open Researcher ID).

The first article introduces various wild food plants (WFPs) consumed in the Mediterranean diet. The expression “wild edible plants or (WEPs)” is used in the title of the first paper, and the term “WFPs” is systematically used in the text. How WFPs are gathered and utilized resembles how WEPs are foraged and consumed in Western countries [1]. The best WEPs have (1) a long history of use, (2) an extensive distribution, and (3) chemical analyses and toxicological research to show that they are safe to consume. Ethnobotanical research is essential to show which species are currently used. They likely have a long history of use if they are used in large geographical areas. This excellent paper, however, has a tiny misprint: the genus *Convolvulus* is written as “*Convulvulus*”.

There are 111 species used in ethnobotanical analyses. This article mainly uses genus-level classification for species; for instance, 2 shows how each WFP genus is consumed. The 111 species are grouped into 70 genera. For foragers, it offers hypotheses to test which species within each genus in their region have undergone chemical and toxicological analyses to confirm their safety for consumption. Some species are widely known as edible weeds. Less known as WEPs are, for instance, *Convolvulus* leaves used in salads and soups and for medicinal purposes; *Crepis* leaves in salads, soups, and stews; *Leontodon* leaves in salads, teas, and as greens; *Tragopogon* leaves in soups and stews and for medicinal purposes; and *Vicia* pods and seeds in soups, stews, and salads.

The essential points for foragers worldwide are as follows: (1) This paper presents evidence supporting the hypothesis that “ancient trade routes influenced the spread and cultural exchange of wild food plant use across the Mediterranean.” Research reports support the view that agriculture started in the Fertile Crescent in the Middle East. Globally, the latest research suggests that agriculture started independently in many distant regions during the Neolithic Revolution about 12,000 years ago [2]. However, different plants were cultivated in different areas. Europeans have spread many cultivated plants and weeds worldwide [1].

Earlier ethnobotanical reports, distribution maps, and direct field observations support the view that the spread of agriculture from Mediterranean countries also led to the spread of edible weeds, such as *Capsella bursa-pastoris*, *Chenopodium album*, *Sonchus arvensis*, *Cirsium arvense*, *Stellaria media*, *Urtica dioica*, etc., worldwide [1]. Edible weeds are a food source when harvest is diminished because of climate change, wars, or other causes. (2) “Recent developments in food security, biodiversity conservation, and sustainable agricultural practices have highlighted the critical role of wild food plants (WFPs) in enhancing ecosystem resilience and ensuring food security in the face of climate change.”

The second article focuses on a Chinese medicinal food plant: *Disporopsis aspersa*. This species exemplifies many local WEPs and their need for chemical analyses. In this case, the results are fascinating. (1) A sustainable method of gathering this plant is explained. (2) Locals have started to grow this plant in their gardens. Cultivation is a sustainable way to prevent overharvesting in nature. (3) In Table 2, *Disporopsis aspersa* is compared to Chinese cabbage (*Brassica rapa*) and Spinach (*Spinacia oleracea*) in terms of the amount of protein, carbohydrate, fat, and dietary fiber each species contains. *Disporopsis aspersa* is revealed to be the more beneficial food plant. The amount of plant protein is impressive, at 27.13 g/100 g, and the share of dietary fiber is high, at 37.73 g/100 g. (4) The mineral content of *Disporopsis aspersa* is excellent. *Disporopsis aspersa* has more potassium (K) and magnesium (Mg) than Chinese cabbage (*Brassica rapa*) and Spinach (*Spinacia oleracea*). *Disporopsis aspersa* also contains more vitamins than Chinese cabbage (*Brassica rapa*) and Spinach (*Spinacia oleracea*). (5) “The findings indicate that *D. aspersa* is an excellent source of amino acids, providing nearly all the amino acids required by humans. The high content of lysine, aspartic acid, and glutamic acid in *D. aspersa* contributes to its umami flavor, while histidine and alanine contribute to its sweetness”. This plant seems to be suitable for international trial cultivation.

The third article is scientifically simpler compared to the other three articles. However, because of its focus on the Catalan linguistic area, it deserves a place in this special issue. Like other languages, Catalan represents a cultural border. This article focuses on Catalan culture and does not observe development outside of this linguistic area. The first article is an excellent introduction to ethnobotanical research on the Mediterranean and neighboring culture and language areas, including Catalan.

This article designates three interesting species as WEPs, such as (1) *Molopospermum peloponnesiacum* (L.) W.D.J.Koch (*Molospermum peloponnesiacum* L.), which is a traditional Catalan wild salad [3]. This plant is suitable for trial cultivation in other regions. (2) Caper bush (*Capparis spinosa)* has potential as a crop plant in dry Mediterranean countries [4]. (3) Young stalks of giant reed (*Arundo donax*) have also been used to make beverages [5].

The fourth article presents innovative research on African wood-sorrel (*Oxalis pes-caprae*). Analyses of this WEP’s proximate composition (sugars, organic acids, minerals, amino acid profile, fatty acid content, and volatile profile) in aerial parts (flowers, leaves, and stalks) provide essential knowledge for the food and pharmaceutical industries. The observations made from the results are as follows: (1) In the article, leaf and flower stalks are called stems. In plant morphology, they are referred to as stalks. In the case of a leaf, the stalk is often called a petiole. These are minor issues in this otherwise excellent article. When the Guest Editor analyses the results, he uses the term “stalk” instead of stem. (2) Stalks have more dietary fiber than flowers and leaves. “Current results highlight that *Oxalis pes-caprae* has higher fiber content than other similar plants”. (3) Leaves have more healthy plant protein than flowers or stalks. (4) The leaves have more healthy fats than flowers or stalks. (5) The leaves and stalks have more ascorbic acid (vitamin C) than the flowers. (6) Comparing macro-mineral results gives interesting results: (a) The flowers and stalks have more potassium (K) than the leaves. (b) The leaves have more magnesium (Mg) than the flowers and stalks. A striking result is that yellow flowers have more magnesium (Mg) than green stalks. This is exciting because magnesium (Mg) is a central atom in chlorophyll and takes part in over 600 enzymatic reactions in human metabolism [6,7,8]. Chlorophylls are green. Stalks are green, but flower petals are bright yellow; only the sepals are green. (c) The leaves have more calcium (Ca) than flowers and stalks. Comparing micro-minerals yields two interesting results: The flowers have more Zinc (Zn) than the leaves and stalks. Secondly, the flowers have more iron (Fe) than the leaves and stalks.

The amino acid analysis found nineteen amino acids. Essential amino acids were the most abundant in flowers, followed by leaves, and the lowest in stalks. One of the non-essential amino acids is glutamic acid; it predominates in the flowers and leaves of *Oxalis pes-caprae*. Glutamic acid is essential for human and plant magnesium (Mg) metabolism. The second article states, “The high content of lysine, aspartic acid, and glutamic acid in *D. aspersa* contributes to its umami flavor, while histidine and alanine contribute to its sweetness.” Four of these amino acids are also in *Oxalis pes-caprae*. Aspartic acid is missing. Glutamic acid content is high, lysine content is low, and alanine content is high. Histidine content is the highest in flowers and the lowest in leaves and stalks.

Plant fatty acids are interesting. In African wood-sorrel (*Oxalis pes-caprae*), there are 29 different fatty acids. Many promote health and longevity. For example, African wood-sorrel (*Oxalis pes-caprae*) has oleic acid, a crucial fatty acid in extra virgin olive oil. As usual in WEPs, African wood-sorrel (*Oxalis pes-caprae*) has more omega-3 polyunsaturated fatty acids than polyunsaturated omega-6 fatty acids. Both promote health. This ratio promotes health, and almost all WEPs have this ratio of fatty acids.

In analyses of volatile substances, 32 compounds were found. The three main volatile substances were nerolidol, caryophyllene, and 3-hexen-1-ol acetate. Nerolidol promotes health in many ways [1]. Caryophyllene promotes health in at least 11 different ways; for instance, it prevents Alzheimer’s disease, according to experimental research [1]. Using Google Scholar and the keywords <3-hexen-1-ol acetate> <health>, it is easy to find that this compound causes a flowery and fruity scent. which is a good attribute in cooking. It is not poisonous. The total content of volatile compounds was significantly higher in *Oxalis pes-caprae* flowers, followed by leaves and stalks. Table 6 shows the volatile compounds found in *Oxalis pes-caprae*. According to the column of scent (“odor”) descriptors, most scents are floral, fruity, herbal, sweet, spicy, balsamic, etc., which are good attributes in cooking. Foragers eat fresh flowers of African wood-sorrel (*Oxalis pes-caprae*).

The authors conclude that “*Oxalis pes-caprae* L. (WEP) is a plant with great future progression due to its nutritional quality since it could be used in the food, nutritional, or pharmaceutical fields”.

The forager’s viewpoint is missing, but it is presented in Åhlberg’s handbook [1]. African wood-sorrel (*Oxalis pes-caprae*) is a traditional Mediterranean edible wild plant [9]. Mediterraneans eat raw African wood-sorrel (*Oxalis pes-caprae*) in salads. The raw leaves and flowers of African wood-sorrel (*Oxalis pes-caprae*) have a fresh, acidic taste. These leaves and flowers can be used as edible, healthy decoration for dishes [1]. African wood-sorrell (*Oxalis pes-caprae*) is edible and, in moderate quantities, unhazardous [10]. It also contains oxalic acid 1148 mg/100 g of fresh leaves, about 1.1 g oxalic acid/100 g of fresh leaves [9]. The level of oxalic acid in the leaves of African wood-sorrell (*Oxalis pes-caprae*) is at the same level as in the leaves of spinach (*Spinacia oleracea*) and the petioles (stalks) of rhubarb (*Rheum rhabarbarum*) [1]. The bright yellow color of *Oxalis pes-caprae*’s flowers is caused by aurones, which are a type of flavonoid. According to experimental research, aurones promote health, such as preventing Alzheimer’s disease [1]. One of the best and safest ways to consume African wood-sorrel (*Oxalis pes-caprae*) is a boiled mixture of WEPs [1]. From the fourth article, foragers learn that the leaves, flowers, and stalks of African wood-sorrel (*Oxalis pes-caprae*) can be used in a boiled mixture of WEPs.

## 3. Conclusions

Both this Editorial and the four articles encourage learning, understanding, and using WEPs to promote health and longevity.

The list of articles accepted for publication:

(1) Alrhmoun, M.; Sulaiman, N.; Pieroni, A. Phylogenetic Perspectives and Ethnobotanical Insights on Wild Edible Plants of the Mediterranean, Middle East, and North Africa. Foods 2025, 14, 465. https://doi.org/10.3390/foods14030465.
(2) Chen, Q.; Wang, M.; Hu, X.; Zhang, J.; Zhang, Q.; Xu, C.; Long, C. Traditional Knowledge and Efficacy Analysis of an Emerging Medicinal Food Plant: Disporopsis aspersa. Foods 2025, 14, 72. https://doi.org/10.3390/foods14010072.
(3) Casas, M.; Vallès, J.; Gras, A., Nutritional Properties of Wild Edible Plants with Traditional Use in the Catalan Linguistic Area: A First Step for Their Relevance in Food Security. Foods 2024, 13, 2785. https://doi.org/10.3390/foods13172785.
(4) Clemente-Villalba, J.; Burló, F.; Hernández, F.; Carbonell-Barrachina, Á.A. Potential Interest of Oxalis pes-caprae L., a Wild Edible Plant, for the Food and Pharmaceutical Industries. Foods 2024, 13, 858. https://doi.org/10.3390/foods13060858.

## Data Availability

Not applicable.
